# Changes in the prevalence, causes of visual impairment, and cataract service coverage in Karamoja, Uganda, between 2015 and 2023: Findings from two cross-sectional surveys

**DOI:** 10.1371/journal.pgph.0006299

**Published:** 2026-04-09

**Authors:** Gladys Atto, Emma Jolley, Stevens Bechange, Susan Kikira, Denis Erima, Ambrose Otim, Beatrice Bako, Robert Mayeku, Elizabeth Apio, Juliet Sentongo, Tesfaye Adera, Anthony Wani, Moses Kasadhakawo

**Affiliations:** 1 Ophthalmology Department, Moroto Regional Referral Hospital, Moroto, Uganda; 2 Sightsavers, Haywards Health, Kampla, United Kingdom; 3 Sightsavers, Uganda country office, Jinja, Uganda; 4 Ophthalmology Department, Jinja Regional Referral Hospital, Jinja, Uganda; 5 Ophthalmology Department, Masaka Regional Referral Hospital, Uganda; 6 Ophthalmology Department, Yumbe Regional Referral Hospital, Yumbe, Uganda; 7 Ophthalmology Department, Fortportal Regional Referral Hospital, Fortportal, Uganda; 8 Benedictine Eye Hospital, Tororo, Uganda; 9 Ophthalmology Department, Soroti Regional Referral Hospital, Soroti, Uganda; 10 Ophthalmology Department, Mulago National Referral Hospital, Kampala, Uganda; Universidad Nacional de Colombia, COLOMBIA

## Abstract

Two rapid assessments of avoidable blindness (RAAB) surveys were conducted in the Karamoja subregion, Uganda, in 2015 and 2023. This paper reports the observed changes in prevalence, causes of visual impairment and coverage of cataract services and examines how the trends differ for males and females. Standard RAAB methodology was used in both studies. Two-stage cluster sampling was used to generate random samples of adults aged over 50 years. Participants underwent a simplified visual acuity (VA) exam, a lens exam, and a posterior segment exam using a direct ophthalmoscope for all participants with presenting VA < 6/18. Data was analyzed using an inbuilt command prtesti, and the regression models were developed using Stata v15 statistical software. In 2015, 3,833 participants were enrolled, and 96.8% (3,727) examined. In 2023, 3,450 were enrolled and 91.6% (3,159) examined. The prevalence of all-cause blindness was 6.0% in 2015 and 4.9% in 2023 (p-value = 0.05). The Cataract Surgical Coverage (CSC) for persons with visual acuity threshold of <3/60, < 6/60, and <6/18 was 41.6%, 34.2% and 20.5% in 2015 and 71.6%, 56.6% and 41.5% respectively, in 2023. The effective Cataract Surgical Coverage (eCSC) for the same vision category was 20.0%, 16.2%, and 9.2% in 2015 and 22.5%, 17.1% and 12.4% in 2023 The eCSC was consistently lower among females than males with the gap widening over the years: 15.9%, 12.1%, and 9.1% in females vs 28.4%, 21.6%, and 15.7% in males respectively. The CSC levels differed across assessments while eCSC values remained low. Targeted interventions are needed to improve post-surgical visual outcomes and ensure equity in access for women and individuals with moderate visual impairment.

## Introduction

The World Report on Vision aims to raise awareness on the global impact of eye conditions and visual impairment and the need to address gaps in data [1].The Rapid Assessment of Avoidable Blindness (RAAB) surveys have been a key source of data for eye health in Uganda, with estimates of blindness and severe visual impairment from cataracts varying across different parts of the country from 1.9%-4.9% [2–5]. Major eye health challenges, such as barriers to accessing services, low cataract surgical coverage, and poor outcomes, remain consistent nationwide [2–5]. These issues are further worsened by a shortage of trained ophthalmologists, limited funding, poor infrastructure, and inadequate equipment [6].

The recently completed eye health system assessment (EHSA) in Uganda, the first of its kind, recognized the progress made by the Ministry of Health but also underscores the scale of these challenges [6]. It observed disparities in the quality of eye care services, with many facilities across regions lacking essential equipment and trained personnel and noted that eye health is often not integrated into primary health care systems, leading to missed opportunities for prevention and treatment [6]. Additionally, it highlighted the absence of comprehensive data on eye health indicators, which has impeded effective planning and monitoring of interventions [6,7]. While the fifth Uganda National Eye Health Strategic Plan aims to improve eye care services [8], efforts to strengthen health systems, increase domestic funding, and leverage technologies such as telemedicine to enhance eye care delivery across the country need to be made.

### The Karamoja subregion

Karamoja subregion has a population of approximately 1.4 million people across 9 districts, covering around 27,000 square kilometres [9]. The region has the lowest literacy rate in the country, limited infrastructure, and many residents follow a nomadic lifestyle, which influences health-seeking behaviours [10–12]. Frequent dry spells contribute to low food security and recurring famines [13–15]. As a result, obtaining food often takes precedence over addressing eye health needs [16].

Despite the persistent challenge of trachoma in the region [17], several innovative programmes have contributed significantly to enhancing access to eye health services. The Coordinated Approach to Community Health (CATCH) programme implemented by the Moroto Regional Referral Hospital between 2015 and 2019 with support from UK foreign, Commonwealth and Development office through UKAID match Grant to Sightsavers, [18] was instrumental in increasing the availability of trachoma eye care treatments and cataract surgical services. Following this, the Karamoja Inclusive Eye Health Programmes (2019–2023) from the same funder continued to build upon these gains by focusing on expanding service provision, strengthening infrastructure, training eye care personnel, supplying ophthalmic equipment, and supporting data management systems.

After the completion of the 2015 RAAB in the subregion, the regional referral hospital benefited from the deployment of its first resident ophthalmologist, who was trained during one of the aforementioned programmes. In addition, the region saw the training of six ophthalmic clinical officers, two for the regional referral hospital and four for the other primary eye care facilities that did not have any eye care workers. Two ophthalmic nurses and one equipment technician were also trained for the regional referral hospital, all aimed at reinforcing the eye health workforce. The trained ophthalmic clinical officers were equipped with essential eye care equipment, enabling them to diagnose and refer a variety of eye conditions effectively.

To further strengthen eye health services, the regional referral hospital in Moroto was provided with necessary infrastructure, ophthalmic equipment, and consumables. This enabled the hospital to maintain and expand its capacity to conduct community cataract outreach activities to ensure an increased Cataract Surgical Coverage. Cataract Surgical Coverage (CSC) is an indicator that measures the number of people in a defined population with operated cataract as a proportion of those having operable plus operated cataract [19]. However, increased service coverage alone does not equate to improved quality of services. Recognizing this, the World Health Organization (WHO) in 2021 emphasized the importance of routine assessment of effective Cataract Surgical Coverage (eCSC), a metric that captures not only the proportion of individuals in need of cataract surgery but those who received it and obtained the desired outcomes [20].

Subsequently, in 2021, the 74th World Health Assembly (WHA) endorsed a 30%-point increase in the eCSC and effective Refractive Error Coverage (eREC) as part of a broader goal for Universal Health Coverage (UHC) [21]. In the same light, the threshold for a good visual acuity outcome was also adjusted from 6/18–6/12 in recognition of the growing evidence of the impact of mild vision impairment [22] Refractive services in the country remain underdeveloped, with only about 10 optometrists in the entire country, indicating a critical need for capacity building in line with the “P” (Personnel) pillar of SPECS (Services, Education, Personnel, Cost, Surveillance) 2030 [23]. Recent studies found that eREC in regions studied was as low as 0.6%–1.0%, emphasizing a large gap to fill by 2030 [23,24].

In alignment with these global priorities, by 2022, the regional referral hospital in Moroto began shifting its focus from merely increasing surgical volume to enhancing the quality of outcomes. This included routine monitoring of surgical outcomes and the introduction of biometry for all cataract patients, even during high-volume outreach services [25]. To understand how the above interventions have affected the prevalence of vision impairment in the region, and to realign services with current needs, a second RAAB was conducted in the subregion in 2023. This paper specifically reports on how the prevalence and causes of visual impairment, as well as both crude and effective coverage of cataract services have changed over the eight-year period. Additionally, the study examines how these trends have varied between men and women over the same period.

## Materials and methods

### Ethics statement

Ethical approval for both studies was granted by the Mulago Hospital Research and Ethics Committee (Ref: MHREC-871 and MHREC-2022–83) and the Uganda National Council for Science and Technology (Ref: SS1558ES). Administrative clearance to conduct the study were obtained from Kotido, Kaabong, Abim, and Moroto district administrations for the 2015 survey and from Nakapiripirit, Nabilatuk, Napak, and Moroto district administrations for the 2023 survey. The community leaders were informed about the survey date and purpose several days in advance of the enumeration team’s visit. On arrival of the team, the area guide, normally a community health worker, was given a translated information sheet which was used to get informed verbal and written consent from the participant. The information provided included the purpose, procedure, and possible benefits of the study.

In both 2015 and 2023, participants were given oral information about the study and provided with an opportunity to ask questions. It was stressed that participation was entirely voluntary, and written consent was taken with adaptations for those who were illiterate or had other impairments preventing them from signing their own name. Participants were paid a token of 1.4 USD to compensate for their time in the 2023 survey, a requirement that was introduced by the Uganda National Council of Science and Technology (UNCST) somewhere between the two surveys. Participants examined and found to have ocular morbidities were either treated on site, given referral to the nearest health facility with required services, or asked for permission to share their name with the team recorder to ensure they would be transported to Moroto Regional Referral Hospital at the next available time.

### Study design

Two cross-sectional surveys using the standardised RAAB methodology [26] were conducted eight years apart in the Karamoja subregion, Northeastern Uganda: the first one in the north from 22/11/2015 to 11/12/2015, and the second in the south from 13/02/2023 to 27/02/2023. Moroto district, being more central, was included in both surveys. Both surveys aimed to assess visual impairment prevalence among individuals aged 50 years and above, identify its causes, and measure CSC and eCSC. There were slight changes introduced in 2023 in VA measurement where the threshold for VA was < 6/12 and also, some of the 2015 data were reanalysed to reflect the 2014 census data, which wasn’t available at that time.

### Sampling

The sampling approach for both surveys followed the standard RAAB approach [26]. Individuals aged 50 years and older residing in the study area were the primary sampling unit. A two-stage sampling process to select them was employed: probability proportional-to-size (PPS) was used to choose enumeration units, villages, followed by compact segment sampling (CSS) within these villages to select individuals.

For both surveys, PPS sampling involved creating a list of all enumeration units in the region based on district-level 2014 census data and checked against more recent village data maintained by district health teams. Population sizes were listed alongside village names, and this information was uploaded into RAAB software which deployed PPS to select the required number of villages. A cluster informer worked two days ahead of the enumeration team to visit the selected villages and provide information to the local leader and eligible population about the study. If the village was sufficiently large, the cluster informer worked with the leader to perform CSS and select a smaller portion of the village for participation in the RAAB.

Sample size calculations were made for both surveys using the calculator built into the RAAB software and were based around the objective of achieving a precise prevalence sample. In 2015, the calculated sample size was 3,833 individuals, organised into 77 clusters of 50 participants each. This determination was made based on an anticipated prevalence of blindness in adults over 50 years of age at 4.0%, with a desired precision of 20% (yielding a minimum acceptable result of 3.6%), 95% confidence intervals, a cluster design effect of 1.5, and an estimated nonresponse rate of 10%.

For 2023, the sample size was estimated at 3,450 individuals, corresponding to 69 clusters of 50 participants each. The calculation took into account a presumed disability prevalence of 10%, alongside an expected prevalence of severe visual impairment of 16% among people with disabilities and 8% among individuals without. A10% nonresponse rate was also factored into the calculation.

### Inclusion and exclusion criteria

Individuals eligible for inclusion in the survey were those aged 50 years or older who had resided in the household for at least the past six months. Persons under the age of 50, as well as those recently residing in the household or visiting, were considered ineligible. Eligible individuals who were absent during the study team’s visit were enrolled but not examined; in such cases, basic information regarding their visual status was obtained from family members or neighbours.

### Data collection procedures

The data collection teams for both surveys were composed of an ophthalmologist and an ophthalmic clinical officer. All team members completed a standardised one-week training programme administered by a RAAB-certified trainer and successfully achieved the minimum acceptable kappa score of 0.6 across nine indicators during the intra-observer variation assessment.

The study procedures remained consistent across both survey periods. Upon arrival at a household, the study team introduced themselves to the head of the household and identified eligible residents for participation. Information regarding the study was shared with eligible individuals, and written informed consent was obtained. Presenting VA was assessed, with spectacles if available, outside the home using a simplified ‘E’ optotype per standard RAAB protocol.

In 2015, participants exhibiting VA < 6/18 in either eye underwent further examination with a pinhole occluder to determine their best-corrected visual acuity. In 2023, the cut-off was VA < 6/12 (for purpose of comparison, this paper focuses on <6/18 threshold), Lens examinations for all participants were performed in a darkened setting, typically within the household, to assess lens status as normal, occluded, aphakic, pseudoaphakic (with or without posterior capsule opacification), or not visible. For eyes presenting with VA < 6/18, the primary cause of visual impairment was determined, including posterior segment examination when indicated. The cause assignment adhered to the standardised RAAB methodology. Individuals with unoperated cataracts were queried regarding barriers to seeking care, while those with prior cataract surgery provided details about their procedures. The definitions used in the study are summarized in Table 1.

**Table 1:** Study definitions.

Term	Definition
**Visual impairment categories** [27]	
Blindness	VA < 3/60 in the better eye with available correction
Severe VI	VA < 6/60 – 3/60 in the better eye with available correction
Moderate VI	VA < 6/18 – 6/60 in the better eye with available correction
**Cataract Surgical Outcome (CSO)** [28]	presenting visual acuity in the operated eye of a person who had undergone unilateral cataract surgery, and presenting visual acuity in the better eye of a person who had undergone bilateral cataract surgery
Good visual outcome	VA ≥ 6/18 in the operated eye
Borderline visual outcome	VA ≥ 6/60 but < 6/18 in the operated eye
Poor visual outcome	VA < 6/60 in the operated eye
**Cataract Surgical Coverage (persons)** [20]	[(x+y)/(x+y+z)]x100 Where: x = persons with unilateral (pseudo) aphakia and an operable cataract in the other eye, y = persons with bilateral (pseudo) aphakia, and z = persons with bilateral operable cataract
**Effective Cataract Surgical Coverage (persons)** [20]	[(c+d)/(x+y+z)]x100 Where: C = individuals with unilateral pseudo/ aphakia achieving presenting visual acuity of 6/18 or better in the operated eye and operable cataract in the other eye D = individuals with bilateral pseudo/ aphakia achieving presenting visual acuity of 6/18 or better in at least one eye and x, y, and z are as above

### Population data

Population data used for both surveys were taken from the 2014 census [11], as shown in Table 2.

**Table 2:** Target population and sample examined in 2015 and 2023 surveys.

2015 Survey participants				Study area population		
	Male	Female	Total	Male	Female	Total
	n (%)	n (%)	n (%)	n (%)	n (%)	n (%)
50-59	494 (34.7)	941 (40.8)	1,435 (38.5)	7,610 (45.6)	10,080 (47.7)	17,690 (46.7)
60-69	435 (30.6)	753 (32.7)	1,188 (31.9)	4,630 (27.7)	5,890 (27.9)	10,520 (27.8)
70-79	346 (24.3)	417 (18.1)	763 (20.5)	2,110 (12.6)	2,510 (11.9)	4,620 (12.2)
80+	148 (10.4)	193 (8.4)	341 (9.1)	2,350 (14.1)	2,660 (12.6)	5,010 (13.2)
**2023 survey participants**				**Study area population**		
50-59	404 (36.5)	579 (28.2)	983 (31.1)	8,470 (38.3)	11,520 (37.5)	19,990 (37.8)
60-69	296 (26.7)	618 (30.1)	914 (28.9)	7,640 (34.5)	12,990 (42.3)	20,630 (39.0)
70-79	263 (23.8)	488 (23.8)	1,014 (23.8)	2,410 (10.9)	3,490 (11.4)	5,900 (11.2)
80 +	144 (13.0)	367 (17.9)	655 (16.2)	3,620 (16.4)	2,720 (8.9)	6,340 (12.0)

### Data analysis

We analysed each survey accounting for the two-stage cluster design. We calculated prevalence estimates and 95% confidence intervals with adjustment for clustering at the cluster level and post-stratification by age and sex using the 2014 census age–sex distribution for the study population. To compare 2015 and 2023 estimates, we used design-based tests of differences in proportions and regression models with survey year as the main exposure, adjusting for age group and sex and using robust variance estimation at the cluster level. Because the number of clusters differed between surveys, inference was based on survey-adjusted standard errors rather than assuming equal cluster structure. For both surveys, descriptive statistics were generated using Stata v18 [29]. A p‑value <0.05 was considered statistically significant.

## Results

The 2015 study enrolled 3,850 people and examined 96.8% (3727 people), while the 2023 survey enrolled 3,450 people and examined 91.6% (3,159 people). In both surveys, participants were predominantly aged 50–59 years, though representation of older age groups improved in 2023 compared to 2015 as shown in Table 2. Women consistently comprised a larger proportion of participants, particularly in the 60–69 and 80 + age groups. In particular, the 2023 survey achieved better inclusion of individuals aged 80 years and above (16.2% vs. 9.1% in 2015).

### Prevalence of visual impairment

Table 3 presents age‑ and sex‑specific prevalence of visual impairment and blindness in the Karamoja region for the 2015 and 2023 surveys. Blindness prevalence was estimated at 6.0% (2015) and 4.9% (2023). Females showed higher blindness prevalence than males, with values of 6.9% across surveys. Severe visual impairment (SVI) prevalence showed limited variation, with estimates of 2.4% and 2.7% in 2015 and 3.0% and 3.2% in 2023 for males and females, respectively. Moderate visual impairment (MVI) prevalence was 9.5% (2015) and 8.0% (2023) overall. Among females, MVI prevalence was 10.3% (2015) and 7.3% (2023). In 2023, female MVI prevalence (7.3%) was lower than that observed among males (10.0%).

**Table 3:** Sample and adjusted prevalence of visual impairment in 2015 and 2023 surveys by age and sex for persons.

	2015			2023			Change	
Males	N	Sample % (95% CI)	Age and sex adjusted (ASA) % (95% CI)	N	Sample % (95% CI)	Age and sex adjusted % (95% CI)	Absolute change in ASA % (95%CI)	p-value
Blindness	72	5.1 (3.8-6.3)	5.0 (3.8-6.4%)	46	4.2 (2.9-5.5)	4.2 (3.1-5.6)	-0.8 (-2.4- + 0.8)	0.34
Severe VI	33	2.3 (1.5-3.1)	2.4 (1.6-3.4)	39	3.5 (2.5-4.5)	3.2 (2.4-4.3)	+0.8 (0.5- + 2.1)	0.22
Moderate VI	141	9.9 (7.9-11.9)	8.9 (7.1-11.1)	102	9.2 (7.4-11.1)	9.1 (7.4-11.1)	+0.2 (2.0- + 2.4)	0.86
**Females**								
Blindness	146	6.3 (5.3-7.4)	6.9 (5.8-8.1)	150	7.3 (5.9-8.7)	5.4 (4.2-6.8)	-1.5 (-2.9- -0.07)	0.04
Severe VI	64	2.8 (1.8-3.7)	2.7 (2.0-3.8)	86	4.2 (3.1-5.3)	3.0 (2.2-3.9)	+0.3 (-0.7- + 1.3)	0.55
Moderate VI	236	10.2 (8.7-11.8)	10.0 (8.6-11.6)	219	10.7 (8.9-12.4)	7.3 (6.1-8.7)	-2.7 (-4.4- -1.0)	0.002
**Total**								
Blindness	218	5.9 (5.1-6.6)	6.0 (5.2-6.9)	196	6.2 (5.1-7.3)	4.9 (4.0-5.9)	-1.1 (-2.2- -0.02)	0.05
Severe VI	97	2.6 (1.9-3.3)	2.6 (2.0-3.3)	125	4.0 (3.1-4.8)	3.1 (2.5-3.8)	+0.5 (-0.2- + 1.3)	0.03
Moderate VI	377	10.0 (8.8-11.4)	9.5 (8.3-10.9)	321	10.2 (8.8-11.5)	8.0 (7.0-9.3)	-1.5 (-2.8- -0.17)	0.03

### Causes of visual impairment

Unoperated cataract was the leading cause of blindness, severe visual impairment (SVI), and moderate visual impairment (MVI) in both surveys, accounting for 43.6% and 39.3% of blindness, 58.8% and 59.2% of SVI, and 46.2% and 42.2% of MVI, respectively as shown in Table 4.

**Table 4:** Causes of visual impairment in the 2015 and 2023 surveys.

	Blindness				Severe visual impairment				Moderate visual impairment			
	2015		2023		2015		2023		2015		2023	
	n	%	n	%	n	%	n	%	n	%	n	%
Unoperated cataract	95	43.8	77	39.3	57	58.8	74	59.2	174	46.2	256	42.2
Trachomatous corneal opacity	57	26.3	23	11.7	6	6.2	3	4.0	25	6.6	5	0.8
Non-trach. corneal opacity	20	9.2	41	20.9	5	5.2	8	8.4	9	2.4	32	5.3
Glaucoma	18	8.3	11	5.6	1	1.0	4	3.2	6	1.6	8	1.3
Other posterior segment/globe diseases	15	6.9	12	8.2	10	10.3	10	8.0	13	3.4	40	6.6
Uncorrected refractive error	4	1.9	2	1.0	8	8.2	8	6.4	142	37.7	197	32.5
Cataract surgical complications	4	1.8	6	3.1	7	7.2	5	4.0	7	1.9	19	3.1
Phthisis	2	0.9	7	3.6	0	0	0	0	0	0	0	0
ARMD	2	0.9	11	5.6	3	3.1	12	9.6	1	0.3	48	7.9
Uncorrected aphakia	0	0	2	1.0	0	0	1	0.8	0	0	1	0.2
**Total**	**217**	**100**	**196**	**100**	**97**	**100**	**125**	**100**	**377**	**100**	**606**	**100**

Corneal opacification was the second most common cause in both surveys. In 2015, trachomatous corneal opacification accounted for 26.0% of blindness, 6.2% of SVI, and 6.6% of MVI. In 2023, corneal opacification due to non‑trachomatous causes accounted for 41.0% of blindness, 8.4% of SVI, and 2.4% of MVI.

Blindness attributable to trachomatous corneal opacification was estimated at 26.3% in 2015 and 11.7% in 2023, while blindness attributed to other causes of corneal opacification was estimated at 9.2% and 20.9%, respectively.

The proportion of SVI attributed to unoperated cataract was 58.8% in 2015 and 59.2% in 2023. Posterior segment diseases also contributed to SVI, including glaucoma and age‑related macular degeneration (ARMD). ARMD accounted for 9.6% of SVI cases in 2023, while other posterior segment conditions accounted for 10.3% in 2015.

### Post-surgical visual outcome

Table 5 compares the visual acuity outcomes by visual acuity levels because in 2015, the threshold for good visual acuity outcome was still 6/18. However, this had been revised to 6/12 by 2023.The postoperative visual acuity outcomes showed similar distributions across assessments. Good outcomes were observed in 42.2% and 39.3% of operated eyes in 2015 and 2023 respectively. Borderline outcomes (visual acuity 6/18–6/60) accounted for 18.0% and 26.4%, while poor outcomes were recorded in 39.1% and 34.3% in 2015 and 2023 respectively.

**Table 5:** Visual acuity outcomes after cataract surgery in 2015 and 2023 surveys.

	WHO norm	2015		2023		% change absolute	p-value
Visual outcome							
Presenting vision	>80%	n	%	n	%		
Good (can see > 6/12)		–	–	71	22.3	-2.9	0.72
Good (can see > 6/18)	<15%	54	42.2	54	17.0		
Borderline (can see > 6/60)	<5%	24	18.8	84	26.4	+7.6	0.45
Poor (can see > 3/60)	<5%	50	39.1	109	34.3	-4.8	0.56
**With pinhole correction**							
Good (can see > 6/12)	>90%	–	–	125	39.3	-0.5	0.95
Good (can see > 6/18)		63	49.2	30	9.4		
Borderline (can see > 6/60)	<5%	25	19.5	69	21.7	+2.2	0.82
Poor (can see > 3/60)	<5%	38	29.7	94	29.6	-0.1	0.99

**Fig 1:**
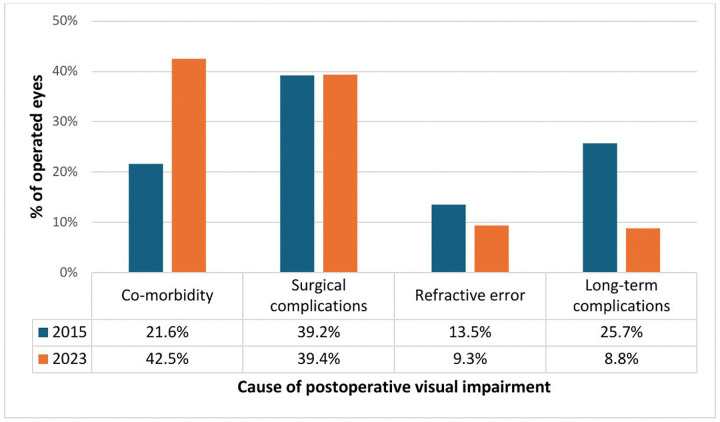
The causes of borderline (>6/60) and poor visual acuity outcomes (>3/60) following cataract surgery in the 2015 and 2023 surveys. Distribution of causes of postoperative visual acuity outcomes classified as borderline (>6/60 to ≤3/60) and poor (>3/60) among operated eyes identified in population‑based surveys conducted in 2015 and 2023. Causes include co‑morbidity, surgical complications, refractive error, and long‑term complications. Bars represent the proportion of affected eyes attributed to each cause, stratified by survey year.

Fig 1 illustrates the causes of borderline (>6/60) and poor visual acuity outcomes (>3/60) following cataract surgery. Ocular co-morbidities emerged as the predominant contributor, accounting for 42.5% of cases in 2023. In contrast, surgical complications were the leading cause in 2015, representing 39.2% of the suboptimal outcomes. Surgical complications remained consistently significant in both years at 39% while long-term postoperative complications played a prominent role in 2015, accounting for 25.7% of cases compared to 8.8% in 2023.

### Cataract surgical coverage (CSC) and effective cataract surgical coverage (eCSC)

Table 6 shows the age- and sex-adjusted CSC and eCSC for males and females in 2015 and 2023. The values of the eCSC across all thresholds of visual impairment: blindness (<3/60), SVI (<6/60), and MVI (<6/18) reveals a generally low coverage in both years. A notable difference was observed only in the < 6/18 category, where eCSC was higher (12.4%) compared with the lower value observed in the corresponding assessment (9.2%; p < 0.001). Among females, eCSC values were lower across all visual acuity thresholds, with the exception of the < 6/18 category, which showed a marginally higher value (9.1% compared with 8.4%).

**Table 6:** Adjusted cataract surgical coverage and effective cataract surgical coverage (persons, percentage) among males and females in 2015 and 2023 surveys.

	2015		2023		Change		Change	
	CSC	eCSC	CSC	eCSC	CSC	p-value	eCSC	p-value
**Males**								
VA < 3/60	46.9	21.5	83.8	28.4	+36.9	<0.001	+6.9	<0.001
VA < 6/60	41.5	18.6	66.7	21.6	+25.2	<0.001	+3.0	0.06
VA < 6/18	24.7	10.2	49.0	15.7	+24.3	<0.001	+5.5	<0.001
VA < 6/12	–	–	42.8	12.8	–	–	–	–
**Females**								
VA < 3/60	37.3	19.0	58.0	15.9	+20.7	<0.001	-3.1	0.007
VA < 6/60	28.8	14.3	45.7	12.1	+16.9	<0.001	-2.2	0.03
VA < 6/18	17.4	8.4	34.1	9.1	+16.7	<0.001	+0.7	0.41
VA < 6/12	–	–	27.0	6.8	–	–	–	–
**Total**								
VA < 3/60	41.6	20.2	71.6	22.5	+30.0	<0.001	+2.3	0.02
VA < 6/60	34.2	16.2	56.6	17.1	+22.4	<0.001	-0.9	0.32
VA < 6/18	20.5	9.2	41.5	12.4	+21.0	<0.001	+3.2	<0.001
VA < 6/12	–	–	34.5	9.7	–	–	–	–

### Barriers to cataract surgery

Table 7 describes reported barriers to cataract surgical services among participants with bilateral cataract and best‑corrected visual acuity <6/60, disaggregated by sex in each survey. Across both surveys, lack of awareness that cataract treatment is possible was the most frequently reported barrier. Inability to access services and the perception that surgery was not needed were also commonly cited. Financial constraints and fear of surgery were reported less frequently, with women more often citing cost‑related barriers than men. Cultural beliefs and provider‑related barriers were uncommon.

**Table 7:** Barriers to cataract surgical services in 2015 and 2023 surveys.

2015 survey			
Barriers	Male	Female	Total
Unaware treatment is possible	20 (50%)	54 (53.5%)	74 (52.5%)
Cannot access treatment	16 (40%)	40 (39.6%)	56 (39.7)
Need not felt	2 (5%)	4 (4%)	6 (4.3%)
Treatment denied by provider	2 (5%)	1 (1%)	3 (2.1%)
Fear	0	2 (2%)	2 (1.4%)
**2023 survey**			
Unaware treatment is possible	17 (39.5%)	44 (37.9)	61 (38.4%)
Need not felt	14 (32.6%)	38 (32.8%)	52 (32.7%)
No access to treatment	9 (20.9%)	17 (14.7%)	26 (16.4%)
Cannot afford operation	1 (2.3%)	7 (6.0%)	8 (5.0%)
Fear of surgery or poor result	1 (2.3%)	5 (4.3%)	6 (3.8%)
Fear of eye removal	1 (2.3%)	2 (1.7%)	3 (1.9%)
Treatment denied by provider	0	2 (1.7%)	2 (1.3%)
Cultural beliefs	0	1 (0.9%)	1 (0.6%)

## Discussion

The age‑ and sex‑adjusted prevalence of blindness was lower in the 2023 survey (4.9%) compared with the 2015 survey (6.0%). However, as the two surveys were conducted in different geographic areas of the Karamoja subregion, these differences should be interpreted cautiously as it may reflect a combination of temporal changes and underlying geographic or contextual differences between the surveyed populations. Despite this positive trend, it is important to recognize that the prevalence of blindness in the region continues to be higher than that observed in the western and central regions of the country [2–4,24]. It is worth noting that, unlike other regions, Karamoja faces significant socioeconomic disparities. Being a semi-arid region, it grapples with persistent poverty, drought, food insecurity, and malnutrition, all of which negatively impact eye health [9,30].

The primary cause of visual impairment in both 2015 and 2023 remains unoperated cataract. This observation is consistent with findings from repeat surveys in other low and middle-income countries, emphasizing the critical role of targeted interventions [31,32]. However, despite these interventions, only a modest reduction in the proportion of blindness attributed to cataract was observed, decreasing from 43.8% in 2015 to 39.3% in 2023. The persistent significance of unoperated cataract at all levels of visual impairment further highlights the ongoing barriers to accessing eye care services in the region. In both surveys, most participants reported being unaware of the availability of cataract surgical services (52.5% in 2015 vs 38.4% in 2023) as the leading barrier.

This finding is consistent with the socio-economic and infrastructural challenges prevalent in the subregion, including poor road networks, widespread poverty, low literacy rates, and limited access to communication technologies such as mobile phones and radios [12]. In addition, older people with visual impairment and other forms of disability tend to be isolated due to stigma [33] These factors collectively hinder the dissemination of health information and impede service uptake.

Furthermore, the shortage of ophthalmologists and allied eye care professionals exacerbates the challenge of community-level awareness and service delivery [6]. Addressing these issues requires a multifaceted approach that goes beyond awareness campaigns. There is an urgent need for systemic reforms aimed at improving education, infrastructure, and healthcare delivery. Additionally, designing contextually appropriate interventions to address known patient-level factors such as low levels of social support, long distances and lack of reliable public transport [33] will go a long way in influencing awareness and service utilization.

Corneal opacification, arising from both trachomatous and non-trachomatous origins, continues to be a significant cause of blindness in both survey periods. While the study reported a decline in trachoma-related corneal opacification since 2015, the overall proportion remains high compared to other regions of the country [2–4,24]. The endemicity of trachoma in the region [17] is compounded by a combination of poor environmental sanitation, persistent poverty, the semi-arid climate, and the nomadic lifestyle [17]. It is therefore imperative to intensify efforts aimed at controlling the infectious and cicatricial stages of the disease through surgery [34] and Mass Drug Administration (MDA) as done in the recent past through cross-border initiatives [17], but much more needs to be done to tackle the barriers of the surgery, which appears to cut across many endemic regions [35,36].

The data also revealed an increase in opacification resulting from other causes. A previous study reported that children of Karamojong ethnicity had a 11.1% likelihood of blindness from corneal opacification [37]. While this study was conducted during a period of low immunization coverage, the region continues to experience recurrent measles outbreaks [38] and drought-induced famine [39], both of which contribute to Vitamin A deficiency, a known risk factor for corneal opacification [40]. It is therefore probable that survivors end up with some form of corneal opacification in adulthood.

The use of traditional eye medications could be another culprit, as reported in other studies across the country [41,42]. As a result, corneal opacification remains a pressing issue that requires ongoing attention. Conducting a study to identify the common causes of corneal opacification unrelated to trachoma in the region will provide data for targeted preventive measures. On the other hand, the need to expand primary eye care services to enable early identification and referral of patients with keratitis cannot be overemphasized. It is also crucial to shift ‘some’ attention toward making corneal transplantation services accessible for patients suffering from corneal scarring.

Diseases affecting the posterior segment of the eye are becoming increasingly important contributors to visual impairment. In 2023, up to 42.5% of poor outcomes were due to ocular morbidities. Similar results have been highlighted in other studies where cataract surgery was performed in eyes with preexisting posterior segment disorders and in those with obscured fundus view due to cataract [43,44].Because these conditions not only influence cataract surgery outcomes, but also independently impair vision, it is essential to broaden the regional focus beyond cataract. This calls for comprehensive strategies to address eye health challenges such as diabetic retinopathy, glaucoma and age-related macular degeneration.

The age‑ and sex‑adjusted prevalence estimates indicated a lower prevalence of blindness among women in the 2023 survey (5.4%) compared with the 2015 survey (6.9%). In both surveys, women consistently had a higher prevalence of blindness than men (6.9% vs. 5.0% in 2015; 5.4% vs. 4.2% in 2023). As the two surveys were conducted in different geographic areas of the Karamoja subregion, these differences should be interpreted cautiously and may reflect contextual or population differences rather than temporal change. Globally, women have been shown to bear a greater cataract burden than men [45]. In this population, however, the disparity may partly reflect the larger proportion of women in the older age as reported in the demographic data. Additionally, cultural and socioeconomic factors play a role: women often serve as primary caregivers and manage domestic and income-generating responsibilities such as cooking, farming, fetching water, and building homes, [46] which can limit their ability to seek timely eye care services [45].

Cataract surgical coverage (CSC) was higher in the 2023 survey than in the 2015 survey for both males and females across all visual‑impairment thresholds. In contrast, effective cataract surgical coverage (eCSC) remained low in both surveys, with consistently lower levels among women compared with men and little variation between survey rounds. Differences in CSC and eCSC should be interpreted cautiously, as geographic and demographic disparities in cataract service coverage are well documented and vary substantially across settings and populations [47] Women, in particular, face reduced access, a disparity largely driven by inequities in health infrastructure, resource allocation, and service availability [20]. This underscores the need for targeted interventions to address gender-based barriers within eye health systems.

Postoperative visual acuity in Karamoja has remained a significant challenge, with minimal change observed in 2015 and 2023. In both years, the proportion of cases achieving good visual outcomes was way below the WHO’s recommended 80% and that of poor outcomes was equally more than 5% [48]. In our setting, eCSC remains constrained, indicating that increases in surgical volume alone did not translate into equivalent gains in effective coverage. Suboptimal outcomes likely reflect a combination of factors, including uncorrected residual refractive error, limited access to post‑operative refraction and spectacles, and insufficient follow‑up to detect and manage complications.

Typically, in our setting, patients are followed up on day one, after one week and at four to six weeks as recommended by WHO [49]. For surgeries conducted at the regional referral, patients are advised to come back for review while for those conducted during outreaches, the ophthalmic clinical officer in that district does the follow up at the health facility nearest to them. But, like other low- and middle-income countries, postoperative follow up in Karamoja faces multifaceted challenges often resulting into high loss-to-follow up rates leading to insufficient follow‑up to detect and manage complications early as well as carry out postoperative refraction [50]. It is worth noting that prior to late 2018, all cataract surgical services in Karamoja were provided through outreach initiatives led by visiting ophthalmologists due to the absence of a resident ophthalmologist. The quality of such surgeries has been questioned before [51]. Further still, most surgeries conducted during that period utilized standard intraocular lenses rather than customized options from biometry [25]. This limitation likely accounts for the minimal improvement observed when assessing outcomes with pinhole correction. A significant shift, however, occurred in 2022 resulting in an improvement in cataract surgical outcomes, when biometry was introduced for all cataract surgeries, including those performed during outreach programs [25].

Since 2015, eye care programs have been running in the region, and tremendous efforts have been made to improve the cataract surgical services. However, with just one resident ophthalmologist, the capacity to provide these services remains limited. Also, given the population and size of Karamoja and the increasing number of the aging population, this only means that the number of blind people is expected to increase [11,12]. Currently, regular surgical services are only available at Moroto Regional Referral Hospital in Moroto district. This means limited access for individuals residing in other districts within Karamoja, which are many kilometers away. To address this gap, regular outreaches must continue to be conducted, but in the long run, there is a need to operationalize and strengthen the capacity of the district hospitals and Health Center Fours across the region to offer cataract surgical services to meet the growing need for eye care services in the region.

This study is subject to several limitations. First, the two RAAB surveys were conducted in different geographic areas of the Karamoja subregion, with the 2015 survey covering predominantly northern districts and the 2023 survey covering predominantly southern districts, although Moroto district was included in both. These districts maybe homogenous in socioeconomic conditions, but access to services, and programmatic exposure may differ. This may have influenced the observed differences in prevalence and service coverage. As a result, comparisons between the two surveys should be interpreted cautiously and cannot be attributed solely to temporal change. Secondly, the surveys used different assumptions in sample size calculations reflecting their primary objectives, which may have affected the precision of estimates across survey years. Thirdly, women were over‑represented in the older age groups, which may have influenced sex‑specific prevalence estimates despite age‑ and sex‑adjustment. Fourthly, the participants in the 2023 survey received a small compensation (USD 1.4) to offset time and participation costs. While this amount was modest, in theory it may have influenced willingness to participate and could have introduced selection bias, however, in reality we do not believe it had much effect because the area is socio-economically homogenous, and the participation was high in both surveys, and actually higher in the earlier one, which indicates that the money did not play a strong role in decision making. Finally, as with all RAAB surveys, the assignment of a single principal cause of visual impairment per eye may underestimate the contribution of co‑existing ocular conditions, and postoperative follow‑up data were limited to presenting visual acuity at the time of survey.

In conclusion, the findings of this study demonstrate measurable advancements in cataract surgical services in the Karamoja region, reflected in lower levels of blindness and improved cataract surgical coverage. These findings highlight the important contribution of eye health programs, particularly in remote and hard‑to‑reach settings such as Karamoja. Nevertheless, substantial challenges persist. Cataract surgical coverage remains insufficient overall, and postoperative visual outcomes do not consistently meet WHO standards. In addition, inequities in access to services, especially affecting women, continue to be evident. These gaps underscore the need to further strengthen and expand cataract surgical services across the region. Policymakers and eye health implementers should prioritize resources and targeted strategies to improve the availability, accessibility, and quality of cataract care.
